# Supplementary material for: Genome-wide synthetic lethality screen of Bam complex-associated genes in Escherichia coli
Source: eLife. 2026 Oct 8;13:RP99955. doi: 10.7554/eLife.99955 (PMC13649257; doi:10.7554/eLife.99955)
Supplement: Peer review history

# Editor report: eLife Assessment

**DOI:** 10.7554/eLife.99955.3.sa0

**Authors:** Ethel Bayer-Santos

**Affiliations:** The University of Texas at Austin United States

**Keywords:** Convincing, Useful

This study presents a systematic analysis of interactions involving components of the outer membrane protein biogenesis machinery in *Escherichia coli*. The authors identify interactions between Bam-associated proteins and several pathways. The evidence supporting the principal conclusions is **convincing**, including several newly identified interactions as well as findings that reinforce previously proposed connections described in the literature. The resulting dataset provides a **useful** resource for future investigations of bacterial envelope homeostasis and the functions of poorly characterized genes.

# Referee report: Reviewer #3 (Public review)

**DOI:** 10.7554/eLife.99955.3.sa1

In this work, Bryant, et al. investigate genetic interactions between non-essential members of the outer membrane protein biogenesis pathway and other genes in the genome using a transposon-directed insertion sequencing (TraDIS) approach in *E. coli* K-12. The authors identify interactions with other components of the envelope including LPS, peptidoglycan, and enterobacterial common antigen biogenesis, and they tie these interactions to specific members of the outer membrane biogenesis pathway. Although many of these interactions are known and have been previously investigated in the field, the study provides several synthetic phenotypes that could be useful for further investigations.

The strengths of the paper include the unbiased, TraDIS approach, and follow up on the interactions observed. The interactions with genes of unknown function also are of interest as they may suggest experiments to find the functions of these genes. Although the paper could better address the relation of its findings to existing literature, the work in the paper is well controlled and the findings will be of interest to the field.

# Author comment: Author response

**DOI:** 10.7554/eLife.99955.3.sa2

**Authors:** Jack Alfred Bryant, Kara A Staunton, Hannah May Doherty, Micheal B Alao, Xuyu Ma, Joanna Morcinek-Orlowska, Emily Goodall, Jessica Gray, Matthew T Milner, Luke Kidger, Charly D Neilson, Jeff A Cole, Felicity de Cogan, Timothy J Knowles, Monika Glinkowska, Danesh Moradigaravand, Ian R Henderson, Manuel Banzhaf

**Affiliations:** University of Nottingham Nottingham United Kingdom; University of Birmingham Birmingham United Kingdom; University of Birmingham Birmingham United Kingdom; University of Birmingham Birmingham United Kingdom; University of Birmingham Birmingham United Kingdom; Department of Bacterial Molecular Genetics, University of Gdańsk Gdańsk Poland; University of Queensland Brisbane Australia; University of Birmingham Birmingham United Kingdom; University of Birmingham Birmingham United Kingdom; University of Nottingham Nottingham United Kingdom; University of Nottingham Nottingham United Kingdom; University of Birmingham Birmingham United Kingdom; University of Nottingham Nottingham United Kingdom; University of Birmingham Birmingham United Kingdom; Department of Bacterial Molecular Genetics, University of Gdańsk Gdańsk Poland; King Abdullah University of Science and Technology Thuwal Saudi Arabia; The University of Queensland Brisbane Australia; Newcastle University Newcastle United Kingdom

The following is the authors’ response to the original reviews.

**Public Reviews:**

**Reviewer #1 (Public Review):**

Weaknesses:

(1) The cutoffs the authors used to define "conditionally essential" mutants are not reported. The results also lack validation for lethality using a titratable system. It would be ideal to validate several genes in each dataset to determine cutoffs (i.e. 5-fold decrease in insertion mutants) for conditional lethality. It was not done (or described) here.

We acknowledge that independent validation using targeted mutants would further strengthen the assignment of synthetic lethality. As the primary aim of this study was the genome-wide identification of genetic interactions associated with loss of fitness in several mutant backgrounds, such validation was beyond the scope of the current work. Our experiments identified hundreds of lethal combinations and we have six datasets; therefore, validation of these interactions is not feasible and is indeed not common for a publication using TraDIS to generate leads for the community to follow up on. However, we already validated some of the hits in our original submission and compared our TraDIS data to some known synthetic-lethal interactions. We have also revised the manuscript to describe all other loci as candidate synthetic-lethal interactions and have highlighted the need for future validation studies in the Discussion.

Regarding the reviewer’s query on thresholds, candidate synthetic-lethal interactions were identified using the tradis_essentiality.R script within the BioTraDIS analytical framework independently on each library: the six mutant backgrounds (DbamB, DbamC, DbamE, DsurA, Dskp, DdegP) and two *E. coli* BW25113 WT reference sets (an "internal" WT replicate sequenced as part of this study, and an "external" WT dataset from a previous study). This classifies each gene as essential, ambiguous, or non-essential for each library based on the bimodal distribution of insertion indices. Synthetic-lethal gene lists were then built by comparing essentiality classifications between each mutant and the WT sets, which were then flagged as shared/not shared with the internal or external WT essential gene lists in Supplementary Table 1. Therefore, a gene was treated as synthetic-lethal in a given mutant when it was called essential in that mutant but not shared with the WT essentiality call. We have clarified this point on line 923 in the Methods section as follows:

“We ran the tradis_essentiality.R script within the BioTraDIS package independently on each library: the six mutant backgrounds (DbamB, DbamC, DbamE, DsurA, Dskp, DdegP) and two *E. coli* BW25113 WT reference sets (an "internal" WT replicate sequenced as part of this study, and an "external" WT dataset from a previous study[95]). This classifies each gene as essential, ambiguous, or non-essential for each library based on the bimodal distribution of insertion indices [30, 34]. Synthetic-lethal gene lists were then built by comparing essentiality classifications between each mutant and the WT sets, which were then flagged as shared/not shared with the internal or external WT essential gene lists in Supplementary Table 1. Therefore, a gene was treated as synthetic-lethal in a given mutant when it was called essential in that mutant but not shared with the WT essentiality call.”

(2) Also, two mutations that both make the cells sick could provide an additive effect (i.e. dapF and BamB), which doesn't necessarily mean the pathways are linked. The authors should revise their wording. They have not shown genetic linkage in some cases.

We revised the text to address this on line 693. However, the bamC mutant demonstrates no significant fitness cost under any of the conditions tested in the manuscript. Therefore, if this is simply an additive effect then it is not clear how this occurs, especially in the case of the dapF, bamC double mutant, and we offer an alternative explanation in the Discussion based on interpretation of the literature.

(3) Mutations throughout the manuscript are not complemented. It would be ideal to add complementation data to show the gene-phenotype relationship is specific.

We thank the reviewers for highlighting this and have complemented the experiments for the bamB-DNA replication link observation as described in response to reviewer 3.

(4) Also, I would argue the term "conditionally essential genes" should be replaced with "synthetically lethal". Strains were compared in the same conditions but with different genetic backgrounds.

We take the reviewer’s point and revised the text throughout.

**Reviewer #2 (Public Review):**

Weaknesses:

(1) An important control in any genetic interaction study is to do complementation tests to demonstrate that the phenotype observed is indeed due to the missing gene under analysis. Although the Keio library was designed to avoid polar effects, it is impossible to predict other undesirable effects of the deletions (hitting of a non-annotated sRNA or RNA stability effects, for example). Thus, before one can safely conclude that a proposed genetic interaction is real, complementation tests should be carried out. This seems particularly important in the case of a new and surprising interaction, such as that between bamB and DNA replication and repair genes.

We thank the reviewers for highlighting this and have provided the complementation experiments for the bamB-DNA replication link observations.

(2) Why not include the suppressor interactions in the work? There are probably plenty, and in principle, they should be as informative as the conditional essential (or synthetic lethal) ones. The only one highlighted in the paper is that between bamB and diaA, since it nicely fits with the synthetic lethal effects with initiation inhibitors seqA and hda. Even if the authors cannot make sense of the suppressor interactions, their inclusion in the paper should make the dataset richer and more valuable to the community.

Due to the nature of the BioTraDIS pipeline, we focused on gene essentiality and so only picked up potential genes that are essential in the parent but become non-essential in the mutants. This misses observations such as that made for diaA, which we hypothesised and checked manually. The data are publicly available for readers to use for their own studies and we have included some notes in Supplementary Table 1 to explain the filtering process along with another tab including the filtered essential gene lists.

(3) The enrichment analysis in Figure 2B deserves some clarification. What is the meaning of gene ratio? How can single genes of a pathway yield an enrichment signal? Why weren’t seqA and hda included in the DNA replication class in 2B?

We thank the reviewer for highlighting this point and realise we did not include a section on this analysis in the Methods section. As such we have included a section on line 935. KEGG pathway enrichment analysis was performed on the conditionally essential gene sets for each mutant background using the enrichKEGG function from the clusterProfiler R package [PMCID: PMC3339379], with the whole *E. coli* K-12 BW25113 genome used as the background gene set. Gene ratio is defined as the proportion of genes within a given conditionally essential gene set that are annotated to a specific KEGG pathway. Enrichment significance was assessed using a hypergeometric test comparing pathway representation within each query gene set to the whole-genome background.

SeqA and Hda were not included in the DNA replication enrichment category because the KEGG enrichment analysis was based on existing KEGG pathway annotations, in which these genes are not assigned to the DNA replication pathway despite their well-established roles in replication initiation control. Considering the revision of the results regarding DNA replication, we feel this does not warrant further changes.

(4) The writing puts too much emphasis on demonstrating that bam lipoproteins and chaperones are specialized instead of fully redundant. However, I have the impression this is a long-settled conclusion in the field, as the manuscript itself describes at several points when reviewing the literature.

We revised the manuscript throughout to reduce this emphasis.

**Reviewer #3 (Public Review):**

In this work, Bryant, et al. investigate genetic interactions between non-essential members of the outer membrane protein biogenesis pathway and other genes in the genome using a transposon-directed insertion sequencing (TraDIS) approach in *E. coli* K-12. The authors identify interactions with other components of the envelope including LPS, peptidoglycan, and enterobacterial common antigen biogenesis, and they tie these interactions to specific members of the outer membrane biogenesis pathway. Although many of these interactions are known and have been previously investigated in the field, the study provides several synthetic phenotypes that could be useful for further investigations.

The strengths of the paper include their unbiased, TraDIS approach, and follow up on the interactions they observe. The interactions with genes of unknown function also are of interest as they may suggest experiments to find the functions of these genes. The largest weakness of this paper is the use of a gene deletion allele for bamB that is known to be polar leading to decreased expression of an essential gene. This largely invalidates all results related to DNA replication. In addition, it is a weakness that the paper does not adequately address its place in the field through discussion of existing results on the interactions they investigate.

The bamB mutant used here has been widely used in several previous studies (Cox et al., 2017, Gunasinghe et al., 2018, Psonis et al., 2019, Storek et al., 2019, Ranava et al. 2021, Steenhuis et al., 2021, Thewasano et al., 2023) with no concern raised and so we appreciate the reviewer’s expertise here and that they highlighted this issue for us to address.

We thank the reviewer for highlighting this issue, as we have now completed complementation experiments for the CRISPRi depletion experiments and found that expression of bamB from a pBAD plasmid does not complement the DbamB strain in which seqA or hda is depleted, but expression of der in this system does complement the phenotype. Therefore, we have revised the title and the text to remove discussion of this potential link to DNA replication. We have included the new results and revised the existing DNA replication related figures as new figures S6-S8 and included a brief discussion of this polar effect in lines 283-314. We are very grateful to the reviewer.
